# The Search for Elusive Structure: A Promiscuous Realist Case for Researching Specific Psychotic Experiences Such as Hallucinations

**DOI:** 10.1093/schbul/sbu044

**Published:** 2014-06-13

**Authors:** Richard P. Bentall

**Affiliations:** ^1^School of Psychological Sciences, Institute of Psychology, Health and Society, University of Liverpool, Liverpool, UK

**Keywords:** hallucinations, diagnosis, schizophrenia

## Abstract

Problems in psychiatric classification have impeded research into psychopathology for more than a century. Here, I briefly review several new approaches to solving this problem, including the internalizing-externalizing-psychosis spectra, the 5-factor model of psychotic symptoms, and the more recent network approach. Researchers and clinicians should probably adopt an attitude of promiscuous realism and assume that a single classification system is unlikely to be effective for all purposes, and that different systems will need to be chosen for research into etiology, public mental health research, and clinical activities. Progress in understanding the risk factors and mechanisms that lead to psychopathology is most likely to be achieved by focusing on specific types of experience or symptoms such as hallucinations.

The past three decades have seen a steady increase in the volume of research on psychotic phenomena. In the early 1980s, publications on symptoms were rare (36 papers with “hallucinations” in their titles were published in 1983 with a cumulative number of 1630 papers until that time; Google Scholar, accessed February 14, 2014). Thirty years later, research on symptoms is more vibrant (336 papers with “hallucinations” in their title published in 2013 with a cumulative total of 6320 papers). This development has been fueled by the invention of new research technologies (notably functional magnetic resonance imaging) that have made symptoms more tractable objects of scientific inquiry, and by a need to understand underlying mechanisms prompted by the emergence of novel interventions. In introducing this Special Supplement on Hallucinations, I want to focus on a third compelling reason for focusing research on particular kinds of psychotic experience such as hallucinations: the apparently intractable problem of psychiatric classification.

## Recent Attempts to Solve Problems of Psychiatric Classification

Toward the end of the first century of schizophrenia research, a few observers noted that broad diagnoses such as schizophrenia were scarcely adequate as independent variables in research because they were defined inconsistently, failed to cleave nature at its joints, and grouped together problems which probably had little in common.^1,2^ At the time this was a minority position. However, the need for a nondiagnosis-based research strategy has become much more widely accepted in the intervening years, most notably among biological researchers such as geneticists,^3^ and pharmacologists,^4^ leading NIMH to recently propose the Research Domain Criteria (RDoC) approach.^5^ This debate has not been resolved by the publication of successive editions of the DSM^6^ and, indeed, seems to have intensified with the publication of DSM-5 which, claims to the contrary notwithstanding, has achieved poor reliability in field trials.^7^

One solution to this problem is to attempt to develop empirically based classification systems. Two proposals appear to be gaining traction at the time of writing. First, analyses of patterns of comorbidity between different diagnoses suggest that the common psychiatric conditions fall into 2 broad spectra of internalizing and externalizing disorders, the former subdividing into anxious misery and fear and the latter including conduct and substance abuse disorders.^8^ A large international study of nonpsychotic DSM-defined disorders^9^ recently found considerable comorbidity within the spectra (eg, individuals who met the criteria for depression—an internalizing disorder—had a high probability of meeting the criteria for generalized anxiety, also an internalizing disorder) but not across the spectra. Interestingly, there was no particular order of acquisition when comorbidity occurred (eg, some people became anxious before they became depressed and vice versa). Recent studies have extended this model to establish that the psychotic disorders appear to form a separate, third spectrum.^10^

Research on the symptoms of psychotic patients, however, has produced a somewhat different picture. Using factor analysis, Liddle^11^ first reported 3 clusters of positive, negative, and cognitive disorganization symptoms, but more recent research has converged on 5 factors by adding depression and mania as separate factors.^12,13^ To add further levels of complexity, some studies have suggested that, superordinate to both the internalizing-externalizing-psychosis spectra^14^ and the 5-factor model of psychosis,^15^ there is a general psychopathology or “P” factor common to all psychiatric disorders, perhaps reflecting neuroticism or emotional instability (this latter type of model is known as a “bifactor model”).

**Fig. 1.**
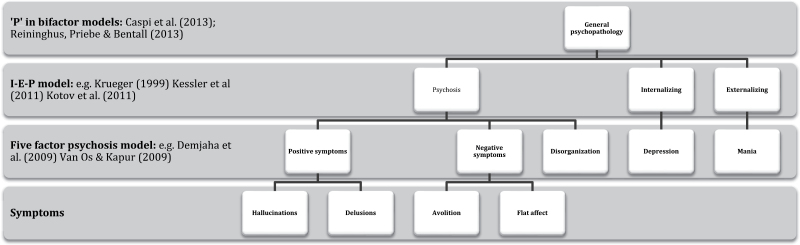
Speculative hierarchical partial classification of psychopathology, integrating the bifactor, internalizing-externalizing-psychosis, and 5-factor models.

It is not yet clear if or how these models can be reconciled. As depression seems to belong to anxious misery in the internalizing spectrum and mania presumably belongs to the externalizing spectrum, it is tempting to nest the remaining psychosis factors within the psychosis spectrum (see figure 1) leading to a hierarchical classification scheme similar to the system used by biologists when classifying species (note that this scheme can, in principle, be continued further, eg, by dividing hallucinations into different subtypes; see McCarthy-Jones et al^16^). However, such a scheme would have to overcome many obstacles. It should be recognized, to begin with, that it is a classification of symptoms and not people (a distinction which is sometimes overlooked). Indeed, patients can obviously experience more than one type of symptom, whichever level we decide to describe them on. There is as yet no evidence that either the Internalizing-Externalizing-Psychosis, 5 factor or hybrid models efficiently predict, eg, responses to particular treatments or map on to etiological factors such as genetic or neuropsychological variables. It is no wonder that many researchers believe that, pragmatically, we have no alternative but to cling to categorical diagnoses for the time being.

Clearly, psychiatric taxonomy is a work in progress, and the comparison with biological species may be instructive. Nearly two and a half centuries after the death of Carl Linnaeus, who devised the general approach to biological classification, some observers have argued that continuing disputes about how to define the boundaries between species cannot be definitively resolved, so that biologists should accept a doctrine of “promiscuous realism.”^17^ Adopting the same doctrine, psychopathologists should perhaps accept that, while there is structure in the way that symptoms co-occur, the structure is so complex that a one size-fits-all method of classification cannot be expected to suit all purposes. What works in etiological research, for the purposes of investigating public mental health and in the clinic may well be different.

## Recognizing Complexity

When a problem seems intractable it is sensible to examine some of the underlying assumptions that have driven past attempts to solve it. Borsboom^18^ reminds us that one such assumption is that syndromes are caused by latent entities. Hence, it is assumed that, if there is a cluster of symptoms corresponding to the diagnosis of schizophrenia, this implies that “schizophrenia” is the cause of the symptoms. This idea leads to the diagnosis being employed as an independent variable when attempting to investigate the etiology of psychiatric disorders. However, structure may arise for many reasons and, as Borsboom points out, is not always best interpreted as evidence of a causal latent entity.

Developing this idea further, Borsboom and Cramer^19^ have proposed that syndromes can arise because symptoms create the conditions in which other symptoms are likely to occur. To take the simplest example possible, sleep problems and problems of concentration both appear in lists of the symptoms of depression but this is not because “depression” (a latent entity inferred from the symptoms) causes both sleep problems and poor concentration but because sleep problems lead to problems of concentration. Moreover, it is also no surprise that depression and anxiety tend to be comorbid diagnoses because problems of concentration are often regarded as symptoms of both. Syndromes and comorbidity between diagnoses therefore emerge as a consequence of networks of causal relations between symptoms.

In an analysis of the positive syndrome which anticipated Borsboom’s work, I previously noted that hallucinations might sometimes give rise to delusions and vice versa.^20^ For example, it has long been recognized that delusional beliefs can be prompted by attempts to explain anomalous experiences^21^ and one well-known psychological model of paranoia gives priority to this mechanism.^22^ It is less recognized that the perceptual judgments of hallucinating patients can be influenced by suggestions affecting their beliefs about what they are likely to experience.^23^ Consistent with this network account, a recent study investigated whether hallucinations precede delusions or vice versa in first episode psychosis, and found that 18.2% of patients experienced delusions only, in 19.5% of cases delusions preceded hallucinations by at least 1 month, and in 16.4% hallucinations preceded delusions.^24^ In the remaining 45.9% both symptoms appeared within the same month (interestingly, no patients who experienced hallucinations alone were recorded).

## Shortcutting the Quest for Elusive Structure

Research targeted at particular symptoms such as hallucinations has long been recognized as a rational strategy for bypassing the unsolved problem of psychiatric classification,^25^ as exemplified by the contributions to this special supplement, none of which focus specifically on “schizophrenia.” Borsboom’s^19^ network analysis provides further impetus for this approach, because the attempt to understand the mechanisms underlying each symptom will help us understand the networks of causal relationships between them. For example, to understand that delusions can sometimes lead to hallucinations it helps to know that the perceptual judgments of hallucinating patients are excessively influenced by their prior beliefs. Further advantages of a symptom-based research strategy have been noted by others, eg, the likelihood of revealing mechanisms that are suitable targets for pharmacological^4^ or psychological^26^ intervention.

However, the comorbidity between symptoms observed in taxonomic research and explained by the network model also raises the hazard that factors attributed to one symptom will be misattributed to a comorbid symptom. Avoiding this hazard requires the use of careful designs and appropriate statistical techniques. For example, a wide range of social adversities are known to increase the risk of psychosis, including childhood trauma and the loss of a parent at an early age but, only by controlling for the comorbidity between symptoms is it possible to show that childhood sexual abuse is a more potent risk factor for hallucinations than paranoid delusions and that, conversely, attachment-threatening events are a greater risk factor for paranoia than hallucinations.^27,28^ Similarly, although abnormal metacognitive beliefs have sometimes been thought to play a causal role in hallucinations,^29^ when controlling for comorbid symptoms, it seems that metacognitive beliefs are implicated in the distress associated with hallucinations rather than their occurrence.^30^

As the contributors to this special supplement demonstrate, further progress in symptom-based research will require multiple methodologies and perspectives, ranging from the humanities,^31^ through psychology (many of the contributions) to the neurosciences.^32^ Attention must be given to phenomenology (as demonstrated by most of the other contributions). For some purposes it will be important to study subtypes,^16^ particular modalities (visual hallucinations having been relatively neglected; Waters et al^33^) or dimensions of experience.^34^ Research must not only focus on patients but also healthy individuals^35^ and should consider special groups such as children^36^ or people living in non-Western cultures.^37^ Undoubtedly, symptom-based research has the potential to enhance our ability to help people who are distressed by their psychotic experiences, either through novel interventions^26^ or by aligning itself with service user and expert-by-experience initiatives.^38^

Symptom-based research is compatible with the NIMH RDoC approach^5^ because the strategy concerns the objects of research whereas RDoC concerns the mechanisms to be investigated. I have argued elsewhere that it has the potential to lead to an entirely satisfactory account of psychopathology^20^ but, in the spirit of promiscuous realism, perhaps I should now say that it might have this potential. Whether, after a second century of research on psychosis, we will still need the kind of categorical diagnoses that have been used so often during the first century seems doubtful, but will only become evident with time.
